# Supplementary material for: Taxonomic analysis of metagenomic data with kASA
Source: Nucleic Acids Res. 2021 Mar 30;49(12):e68. doi: 10.1093/nar/gkab200 (PMC8266618; doi:10.1093/nar/gkab200)
Supplement: gkab200_Supplemental_Files [file gkab200_supplemental_files.zip › Suppl_D.zip!/OPAL_comparison_new/results.html]

OPAL: Open-community Profiling Assessment tooL
